# The Utilisation of INR to identify coagulopathy in burn patients

**DOI:** 10.1371/journal.pone.0278658

**Published:** 2024-02-23

**Authors:** Kendall Wermine, Juquan Song, Sunny Gotewal, Lyndon Huang, Kassandra Corona, Shelby Bagby, Elvia Villarreal, Shivan Chokshi, Tsola Efejuku, Jasmine Chaij, Alejandro Joglar, Nicholas J. Iglesias, Phillip Keys, Giovanna De La Tejera, Georgiy Golovko, Amina El Ayadi, Steven E. Wolf

**Affiliations:** 1 School of Medicine, University of Texas Medical Branch, Galveston, Texas, United States of America; 2 Department of Surgery, University of Texas Medical Branch, Galveston, Texas, United States of America; 3 Department of Pharmacology, University of Texas Medical Branch, Galveston, Texas, United States of America; Uniformed Services University of the Health Sciences, UNITED STATES

## Abstract

Studies conflict on the significance of burn-induced coagulopathy. We posit that burn-induced coagulopathy is associated with injury severity in burns. Our purpose was to characterize coagulopathy profiles in burns and determine relationships between % total burn surface area (TBSA) burned and coagulopathy using the International Normalized Ratio (INR). Burned patients with INR values were identified in the TriNetX database and analyzed by %TBSA burned. Patients with history of transfusions, chronic hepatic failure, and those on anticoagulant medications were excluded. Interquartile ranges for INR in the burned study population were 1.2 (1.0–1.4). An INR of ≥ 1.5 was used to represent those with burn-induced coagulopathy as it fell outside the 3rd quartile. The population was stratified into subgroups using INR levels <1.5 or ≥1.5 on the day of injury. Data are average ± SD analyzed using chi-square; p < .05 was considered significant. There were 7,364 burned patients identified with INR <1.5, and 635 had INR ≥1.5. Comparing TBSA burned groups, burn-induced coagulopathy significantly increased in those with ≥20% TBSA; p = .048 at 20–29% TBSA, p = .0005 at 30–39% TBSA, and p < .0001 for 40% TBSA and above. Age played a significant factor with average age for those with burn-induced coagulopathy 59 ± 21.5 years and 46 ± 21.8 for those without (p < .0001). After matching for age, TBSA, and demographics, the risk of 28 day-mortality was higher in those with burn-induced coagulopathy compared to those without (risk difference 20.9%, p < .0001) and the odd ratio with 95% CI is 4.45 (3.399–5.825). Investigation of conditions associated with burn-induced coagulopathy showed the effect of heart diseases to be significant; 53% of patients with burn-induced coagulopathy had hypertension (p < .0001). Burn-induced coagulopathy increases with %TBSA burned. The information gained firmly reflects a link between %TBSA and burn-induced coagulopathy, which could be useful in prognosis and treatment decisions.

## Introduction

Studies conflict on the significance of burn-induced coagulopathy. The first English language publication addressing coagulopathy in burn patients comes from Holder et al. [1] in 1963. This publication led other researchers to investigate the association using murine models, allowing for results with limited generalizability [2]. These studies have persisted throughout the years with conflicting conclusions.

In 2013, Sherren et al. [3] defined the effects of clotting changes caused by thermal injury in a retrospective review of acute burn-induced coagulopathy, using the criteria for coagulopathy of prothrombin time (PT) >14.6 s, activated partial thromboplastin time (aPTT) >45 s, or international normalized ratio (INR) >1.2; 39.3% of severely burned patients met their established criteria upon admission. The study found a correlation between PT elevation and burn severity in addition to demonstrating the use of acute burn-induced coagulopathy as a significant predictor of 28-day mortality after burn injury [3]. In the same year, a study by Lu et al. [4] found no relation between acute coagulopathy and major burn injury. The study’s criteria for acute coagulopathy consisted of an INR ≥1.3, an aPTT ≥1.5 s, and a normal platelet count. A later study from 2016 defined acute burn-induced coagulopathy as an INR >1.5 and an aPTT >60 s; severe burns were defined as ≥30% total body surface area (TBSA) burned. The study found that severe burns are associated with greater coagulopathy with earlier onset but concluded that further investigation was indicated to diagnose and treat burn-induced coagulopathy [5]. A separate study in 2016 surveyed 55 burn ICU physicians and found results varied as some used PT, aPTT, fibrinogen, or platelets as tests to detect burn-induced coagulopathy, demonstrating no diagnostic criteria consensus [2,6]. Our study then aims to further investigate burn-induced coagulopathy associated with burn injury severity via the large TriNetX electronic medical record database. Our purpose was to characterize coagulopathy profiles in patients with burn injury and determine relationships between %TBSA burned and coagulopathy using INR.

## Methods

Data was obtained from the TriNetX database, a North American federated health research network providing global access to electronic medical records from over 69 million patients in 51 healthcare organizations. TriNetX, LLC is compliant with the Health Insurance Portability and Accountability Act (HIPAA), the US federal law which protects the privacy and security of healthcare data, and any additional data privacy regulations applicable to the contributing HCO. TriNetX is certified to the ISO 27001:2013 standard and maintains an Information Security Management System (ISMS) to ensure the protection of the healthcare data it has access to and to meet the requirements of the HIPAA Security Rule. The TriNetX Platform, only contains de-identified data as per the de-identification standard defined in Section §164.514(a) of the HIPAA Privacy Rule. The process by which the data is de-identified is attested to through a formal determination by a qualified expert as defined in Section §164.514(b)(1) of the HIPAA Privacy Rule. Because this study used only de-identified patient records and did not involve the collection, use, or transmittal of individually identifiable data, this study was exempted from Institutional Review Board approval.

Patients with burns were identified in the TriNetX database from 40 healthcare organizations in the United States and analyzed by percent TBSA burned using the ICD-10 codes T31.0-T31.9, which includes patients with burns involving less than 10% of body surface to patients with burns involving 90% or more of body surface. The data was gathered in January 2021 and encompasses all the information TriNetX had gathered over the years. Patients with prior transfusions, chronic hepatic failure, and those on anticoagulant medications were excluded through the TriNetX codes 302, K72.1, and BL110, respectively. Interquartile range (IQR) for the population burned were determined using the TriNetX database analysis tool and the interquartile range was 1.2 (1.0–1.4). An INR of ≥ 1.5 then represents those with burn-induced coagulopathy as being outliers on the distribution outside the third quartile, which is supported by previous studies [3,4]. The population was stratified into subgroups using INR levels <1.5 or ≥1.5 on the day of injury by the TriNetX code 9032. The INR used was taken on the day of injury/admission before any operations had been done. TriNetX is unable to provide hour by hour data for labs in order to keep patient confidentiality, so day of injury/admission was used. The risk of burn-induced coagulopathy in relation to percent TBSA was analyzed by stratifying the patient population in 10% intervals from 0% to 99% TBSA. The population was further stratified by age at injury and ethnicity. In addition, demographic data was analyzed to assess for the incidence of burn-induced coagulopathy. Moreover, 30-day mortality, laboratory values, and diseases associated with burn-induced coagulopathy were also investigated through comparing patients with burn induced coagulopathy and those without using the TriNetX database. The TriNetX analysis tool was used to determine Data mean ± SD which was then analyzed using chi-square following with propensity matching for the analyzed factors; p < .05 was considered significant. TriNetX platform utilizes input matrices of user identified covariates and conducts logistic regression analysis to obtain propensity scores for individual subjects.

We investigated INR as it is a principal indicator of acute traumatic coagulopathy commonly used in medical practice. IQR for our study population was 1.2 (1.0–1.4), therefore, we established this limit for further study of burn-induced coagulopathy upon presentation to the burn center. Our decision for an INR ≥1.5 to indicate burn-induced coagulopathy is supported by the population’s IQR of 1.2 (1–1.4) as an INR of 1.5 falls outside the third quartile. Prior studies found that an INR ≥1.5 was significantly associated with all-cause death, hemorrhagic shock-associated death, venous thromboembolism, and multiple organ failure [7]. Additionally, an INR >1.2 was not associated with an increased risk for the studied outcomes [7]. Using INR ≥1.5 as our criteria for burn induced coagulopathy, we posit that burn-induced coagulopathy is associated with increased injury severity in burns.

## Results

**Fig 1:**
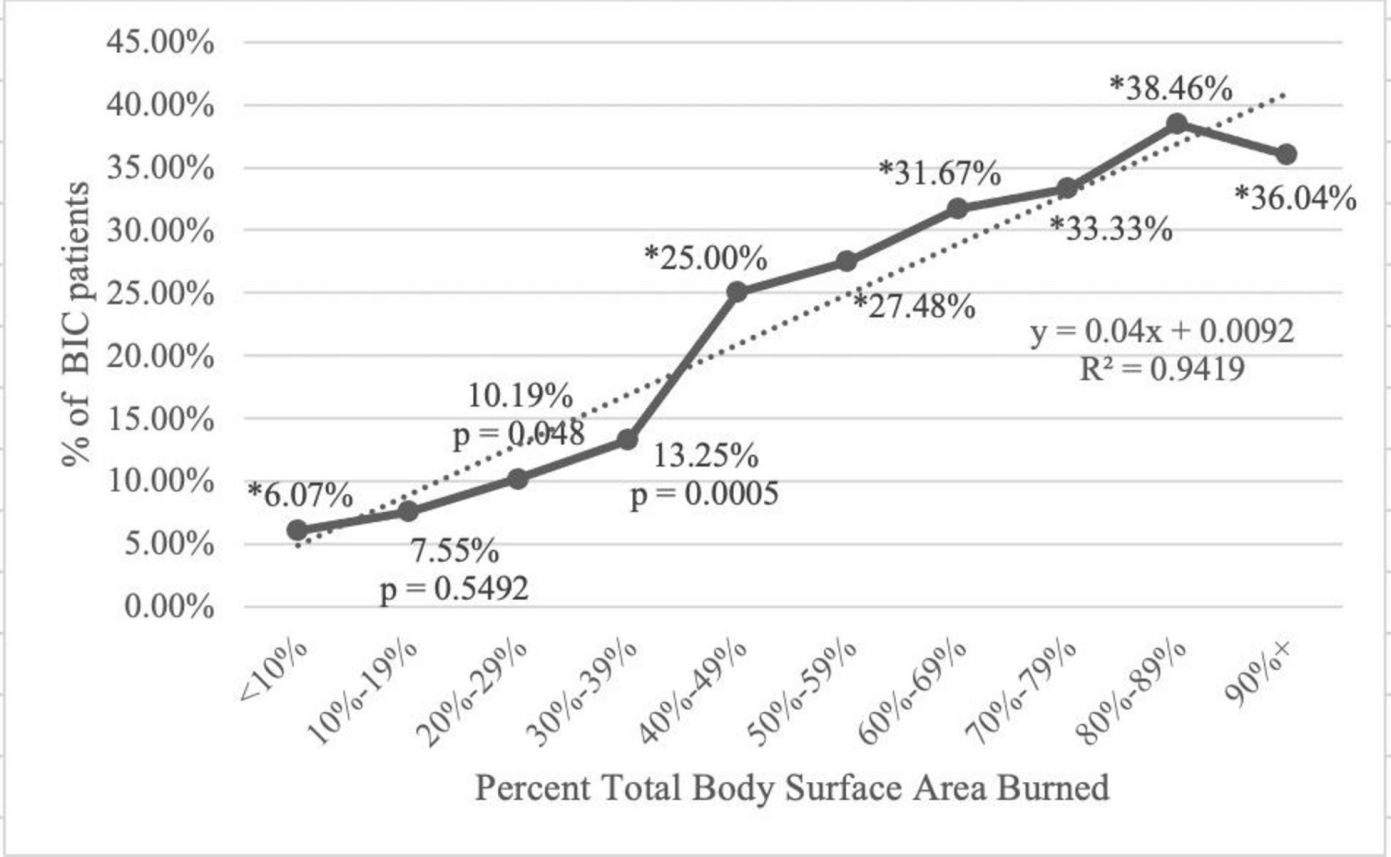
%TBSA and % of burn-induced coagulopathy patients (635 patients total), asterisks indicate p<0.0001.

**Fig 2:**
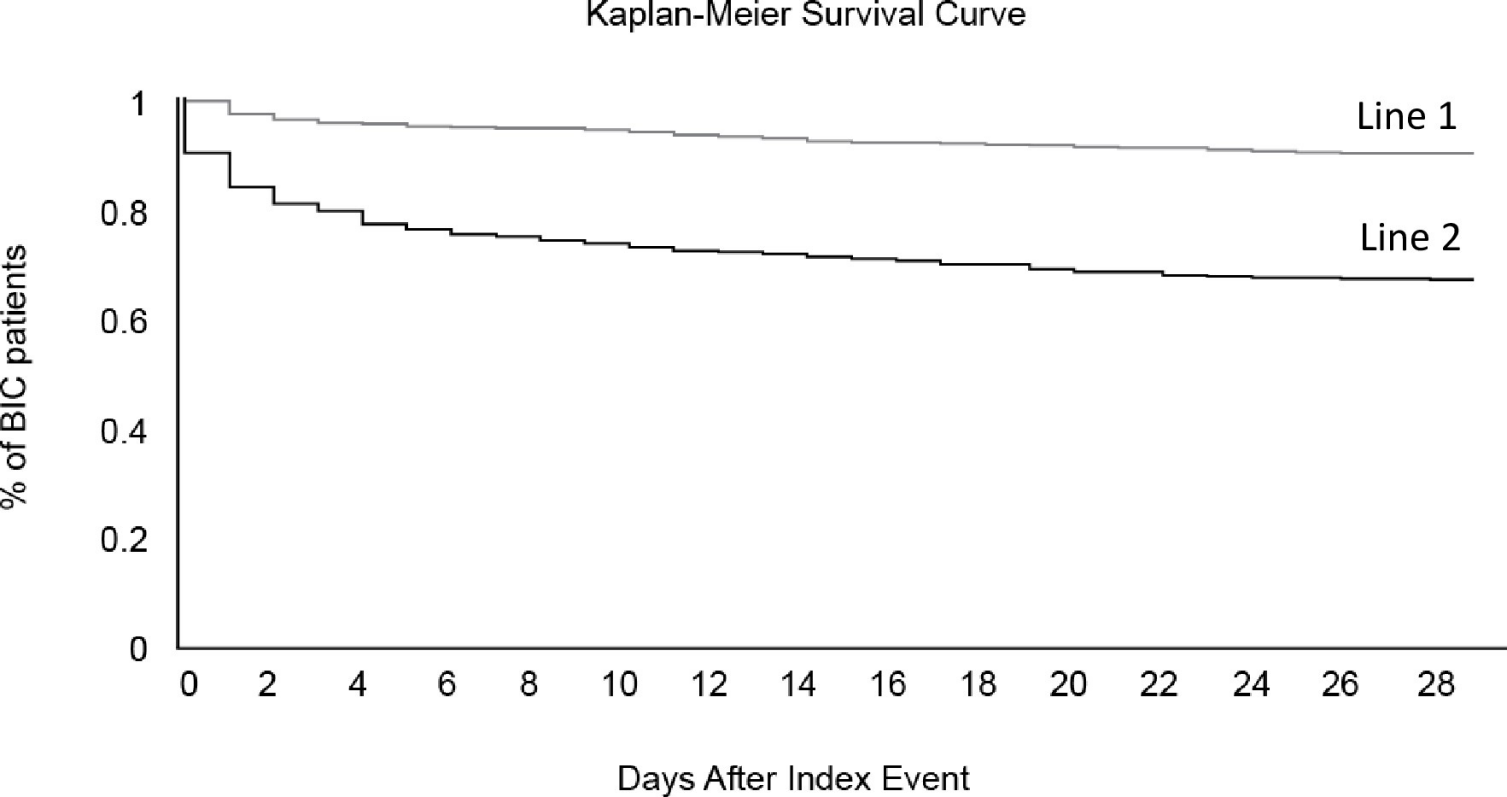
Kaplan Meier Survival Curve showing the risk of 28 day-mortality in patients with burn-induced coagulopathy (line 1) compared to those with an INR <1.5 (line 2), Risk difference is 20.9% (p < .0001).

From 40 of the healthcare organizations in the network spanning the last twenty years, 7,364 burned patients with INR <1.5 and 635 burned patients with INR ≥1.5 at the time of admission were identified using the TriNetX database. The average INR for each group was 1.95 ± 1.23 in patients with burn-induced coagulopathy compared to 1.15 ± 0.47 for those without burn-induced coagulopathy. INR for patients with burn-induced coagulopathy (1.95 ± 1.23) fell into the fourth quartile range of INR for the population 1.2 (1.0–1.4). When groups were compared by %TBSA burned, burn-induced coagulopathy was increased in those with >20% TBSA burned or greater; p = .048 at 20–29% TBSA, p = .0005 at 30–39% TBSA, and p < .0001 for 40% TBSA and above (Fig 1 and Table 1). In addition to propensity matching with patient basic demographics including age, gender, race, we also propensity matched injury severity and TBSA burned to study the non-survivals in burn patients with higher INR. After propensity matching of the baseline characteristics and TBSA burned the odd ratio with 95% CI is 4.45 (3.399–5.825). In addition, using the raw data, the risk of 28 day-mortality was higher in patients with burn-induced coagulopathy compared to those with an INR <1.5 (risk difference 20.9%, p < .0001) as seen in the Kaplan-Meier Survival Curve (Fig 2).

**Table 1:** Percent of BIC patients by TBSA.

% TBSA	Total Patients	% of patients with BIC
<10%	6,080	6.00%
10–19%	1,471	7.50%
20–29%	520	10.20%
30–39%	302	13.25%
40–49%	224	25.00%
50–59%	131	27.50%
60–69%	120	31.60%
70–79%	72	33.30%
80–89%	78	38.50%
90%<	111	36.00%

When considering demographic data for those with burn-induced coagulopathy, the incidence was lower in men (p = .0019) and higher in women (p < .0001). Age was also found to be a significant factor in the development of burn-induced coagulopathy. The average age for patients with burn-induced coagulopathy and patients without burn-induced coagulopathy were 59 ± 21.5 years and 46 ± 21.8, respectively (p < .0001). Stratification by race revealed African Americans were significantly less likely to experience burn-induced coagulopathy (p = .0324); however, we found no association for European-American patients with or without burn-induced coagulopathy even though making up 70% of both cohorts (p = .9880).

**Fig 3:**
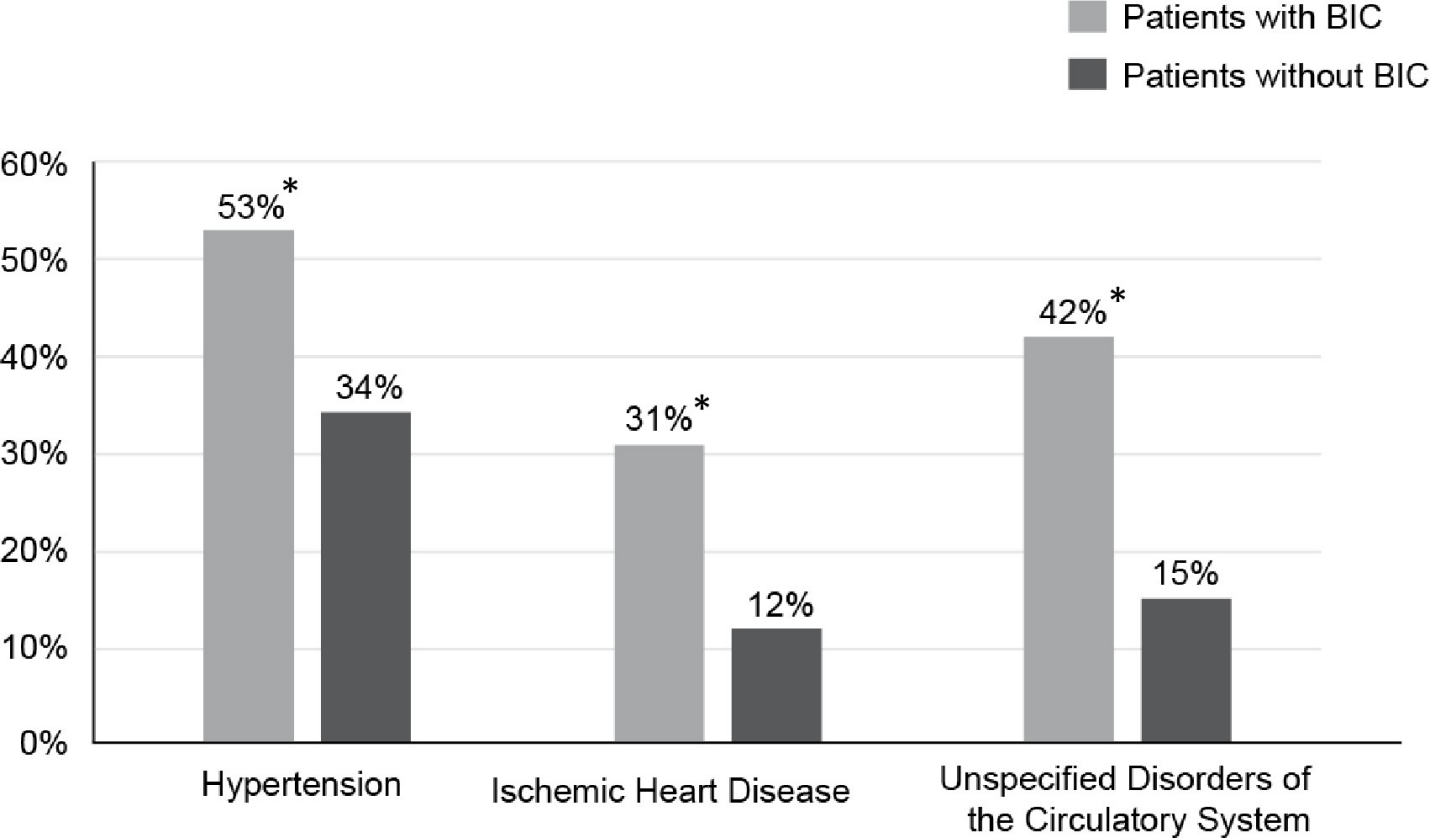
Differences in cardiovascular diseases between patients with burn-induced coagulopathy compared to patients without burn-induced coagulopathy. An * indicates a p-value < .05.

Investigation of conditions associated with burn-induced coagulopathy showed heart diseases to be significant (Fig 3), as ischemic heart disease was present in 31% of burn-induced coagulopathy patients and only in 12% for those without burn-induced coagulopathy (p < .0001). Hypertension also proved to be a significant factor as 53% of patients with burn-induced coagulopathy had hypertension compared to 34% of those without burn-induced coagulopathy (p < .0001). Furthermore, 42% of burn-induced coagulopathy patients had other and unspecified disorders of the circulatory system (ICD-10 code I95-I99), while this was present in only 15% of those without burn-induced coagulopathy (p < .0001).

After excluding chronic hepatic disease, both alanine aminotransferase and aspartate aminotransferase levels were significantly increased (p < .0001) in patients with burn-induced coagulopathy (129 IU/L ± 524; 210 U/L ± 1,127) compared to only mild elevations in those without burn-induced coagulopathy (50.9 IU/L ± 224; 71.8 U/L ± 581). Normal range for alanine aminotransferase and aspartate aminotransferase levels are 22 IU/L (15–33) and 25 U/L (18–35). Cirrhotic patients were then identified using ICD-10 codes K74 and K70.3 and excluded from the data to determine if there was a significant difference. When excluding cirrhotic patients from those with burn induced coagulopathy and reassessing alanine aminotransferase and aspartate aminotransferase levels there was no significant difference; p = .7577 and p = .8367, respectively.

## Discussion

Burn-induced coagulopathy has historically been defined as INR ≥1.5 on the day of injury. Prior studies on acute traumatic coagulopathy supported the decision for an INR ≥1.5 as mentioned in the introduction [7]. Although INR criteria differed from other studies which used an INR > 1.2, [3] we observed a similar increase in mortality. The risk of 30 day-mortality in our study was higher in patients with burn-induced coagulopathy compared to those with an INR <1.5 (p< 0.05), this was also noted in other studies which investigated INR as a predictor of mortality in those with sepsis which found increased mortality in patients with an INR >1.5 [8].

Risk for burn-induced coagulopathy increased with %TBSA burned. Occurrence of severe burn-induced coagulopathy increased in those with ≥20% TBSA burned with the p-value increasing in significance as %TBSA burned increased. The increase in significance when investigating the relationship between %TBSA burned and burn-induced coagulopathy confirms the association of burn-induced coagulopathy with injury severity in burns, especially in severe burn. This is widely thought to be a result of hemodilution caused by resuscitation fluid infusion coupled with hypothermia, which is often seen in patients with severe burns that cover a large surface area [5]. The extent of hemostatic change seems to be generally associated with burn severity; patients with severe burns are thus more likely to develop extensive coagulopathy [5]. The coagulopathy seen in result of burn injury could also be explained by systemic inflammation in response to endothelial damage from the burn. Systemic inflammation results in elevated levels of plasminogen activator inhibitor ultimately decreasing fibrinolytic activity and increasing coagulation [9,10]. Pro-inflammatory cytokines like IL-6 increase both platelet count and platelet responsiveness to thrombin [9,10]. After surviving the initial acute phase of burn recovery, coagulation status is susceptible to many risk factors that come with later recoveries such as infections, sepsis, impaired mobility, and physiological stress [2].

When considering epidemiology, other publications that studied trauma-induced coagulopathy found the population to be 75.3% male, 25.7% of whom have the mean age of 39 years and an overall mortality of 7.3% [11]. Our study of burn-induced coagulopathy also found men to be more susceptible, making up 65% of the affected population. However, women were more likely to have burn-induced coagulopathy when compared to the total population of burn injury patients. Age factored into a patient’s susceptibility as older patients were more likely to develop burn-induced coagulopathy. The effect of age and gender on outcome could be explained by the fact that both women and the elderly are more likely to have preexisting medical conditions which ultimately lead to more severity and worse outcomes of burn patients [12]. Elderly patients are particularly at risk because early excision and skin grafts can result in more blood loss and intubation and anesthesia may not be tolerated well [12]. Compared with patients without burn-induced coagulopathy (46.2 years old), those that developed burn-induced coagulopathy was an average of 13 years older (59.3). Despite a wide range of patient ages (1–90 and 0–90, respectively), we believe that the patient population in both groups is large enough to account for any outliers. Healthcare outcomes based on race seem to play a minor role in the development of burn-induced coagulopathy with African Americans significantly less likely than other races to experience burn-induced coagulopathy (p = .0324) and European American patients showing no significant difference between the groups (p = .9880).

To investigate the role of underlying conditions in the development of burn-induced coagulopathy, we excluded patients with prior transfusions, chronic hepatic failure, and those on anticoagulant medications, as these conditions can alter a patient’s hemostasis [2]. After these parameters were excluded, we found an association between burn-induced coagulopathy and heart disease on the day of admission, a significant period of clinical interest in diagnosis, prognosis, and operative planning. Burn-induced coagulopathy was significantly more prevalent in patients with hypertension and other unspecified disorders of the circulatory system when compared to patients without burn-induced coagulopathy. The impact of preexisting diseases such as cardiovascular, pulmonary, and renal disease make fluid resuscitation remarkably more complicated in the early stages of acute burn management [13]. Lab values of alanine aminotransferase and aspartate aminotransferase were considerably elevated in patients with burn-induced coagulopathy compared to only mild elevations in those without burn-induced coagulopathy (p < .0001). These elevations were not due to other liver diseases, as patients with chronic hepatic failure were excluded from the study. We also investigated the effect of cirrhosis, as it was not excluded from the patient population under the chronic hepatic failure ICD-10 code and found the p-value to exceed .05. The absence of a significant difference implies the diagnosis of cirrhosis is not a confounding variable for the elevation of alanine aminotransferase and aspartate aminotransferase levels in those with burn induced coagulopathy. Elevated liver enzymes in burn-induced coagulopathy are understandable as the liver contributes to both primary and secondary hemostasis by synthesizing coagulation factors [2].

In particular, studies of perfused livers and cultured primary hepatocytes provide direct evidence for the synthesis of coagulation factors by the liver. Sinusoidal and extrahepatic endothelial cells are to be responsible for factor VIII production and endothelial cells specifically secrete von Willebrand factor, which stabilizes factor VIII and captures platelets (Olson 1966, Owen 1977) [12,14]. The liver has an important role in coagulation as indicated by the above studies, which is why we excluded those with liver disease from this study. Including those with liver disease had the potential to be a confounding variable so it was excluded using the ICD-10 code K72.1. We agree with past studies that liver disease is an important factor in coagulopathy disorders, but the scope of our investigation is to determine if there is a connection between %TBSA and burn induced coagulopathy without confounding factors such as liver disease.

The TrinetX registry is newer and covers data at different points in time, which could lead to discrepancies in reporting in different decades, by requiring INR to be taken on the day of injury this problem is mitigated as INR has been a standard of coagulopathy since the 1980s. In recent times, thromboelastography has emerged as an alternative to INR in the assessment of coagulation and coagulopathy following traumatic injury, however thromboelastography was not a data point available in our study but we plan to characterize it in the future. Another topic we plan to investigate in the future is inhalation injury and its relation to coagulopathy. In the future we plan to further separate the data by burn degree and investigate the impact of total necrotic tissue load on burn induced coagulopathy rates in those with higher degrees of burn.

This study has potential limitations given the nature of the TriNetX database. One such limitation is the inability of the database to assess for fluid resuscitation and the lack of resolution provided by the database on initial blood draws. Our study examined INR utilization in the diagnosis of burn induced coagulopathy. INR is not the typical tool used to identify burn induced coagulopathy with the newer technology of functional assays of coagulation, such as thromboelastography, that provide a more complete picture of clot initiation, propagation, and fibrinolysis which could be investigated in future studies. However even with these qualifications we believe the use of the TriNetX database to assess INR use as a surrogate of coagulopathy in identifying burn induced coagulopathy to produced accurate conclusions.

Our observational results were confirmed by running the test again at a later date. A processing data report was created by re-collecting data at 11/21/2023 by TriNetX database analytic program. [**S1 File**] Following the same queries, Group 1 included patients with burn-induced coagulopathy, and group 2 included those without burn-induced coagulopathy. For this additional analysis, INR at 1.5 was set as the threshold of burn induced coagulopathy as done previously. Four 30-day outcomes were assessed including death, hypertension, ischemic heart diseases, and circulatory system disorders with risk ratios and odds ratios. Since data are continuously accumulated and aggregated in TriNetX database, the data in supplemental file do not exactly match the previous data. However, the outcome and conclusions are the same, indicating that those with burn-induced coagulopathy identified by our criteria had higher risks of death and other circulatory system disorder diagnoses in the first 30 days after injury. The supplemental information supports the original data displayed in **Figs 2** and **3** in the manuscript, which was collected in January 2021.

Our study supports our original postulate that burn-induced coagulopathy is associated with injury severity in patients with burns. Burn-induced coagulopathy increases with %TBSA burned, is more prevalent in women, and is influenced by prior disease especially those of the heart. Overall, the information gained from this large database reflects a link between %TBSA burned and burn-induced coagulopathy, which can be utilized in assessment of prognosis and treatment determinations.

## Supporting information

S1 File (DOCX)
